# The impact of the synergistic effect of SO_2_ and PM_2.5_/PM_10_ on obstructive lung disease in subtropical Taiwan

**DOI:** 10.3389/fpubh.2023.1229820

**Published:** 2023-09-22

**Authors:** Te-Yu Chen, Szu-Chia Chen, Chih-Wen Wang, Hung-Pin Tu, Pei-Shih Chen, Stephen Chu-Sung Hu, Chiu-Hui Li, Da-Wei Wu, Chih-Hsing Hung, Chao-Hung Kuo

**Affiliations:** ^1^School of Post-baccalaureate Medicine, College of Medicine, Kaohsiung Medical University, Kaohsiung, Taiwan; ^2^Division of Nephrology, Department of Internal Medicine, Kaohsiung Medical University Hospital, Kaohsiung Medical University, Kaohsiung, Taiwan; ^3^Faculty of Medicine, College of Medicine, Kaohsiung Medical University, Kaohsiung, Taiwan; ^4^Department of Internal Medicine, Kaohsiung Municipal Siaogang Hospital, Kaohsiung Medical University, Kaohsiung, Taiwan; ^5^Research Center for Precision Environmental Medicine, Kaohsiung Medical University, Kaohsiung, Taiwan; ^6^Division of Hepatobiliary, Department of Internal Medicine, Kaohsiung Medical University Hospital, Kaohsiung Medical University, Kaohsiung, Taiwan; ^7^Department of Public Health and Environmental Medicine, School of Medicine, College of Medicine, Kaohsiung Medical University, Kaohsiung, Taiwan; ^8^Department of Public Health, College of Health Sciences, Kaohsiung Medical University, Kaohsiung, Taiwan; ^9^Institute of Environmental Engineering, College of Engineering, National Sun Yat-Sen University, Kaohsiung, Taiwan; ^10^Department of Medical Research, Kaohsiung Medical University Hospital, Kaohsiung, Taiwan; ^11^Department of Dermatology, Kaohsiung Medical University Hospital, Kaohsiung, Taiwan; ^12^Department of Dermatology, College of Medicine, Kaohsiung Medical University, Kaohsiung, Taiwan; ^13^Doctoral Degree Program, Department of International Business, National Kaohsiung University of Science and Technology, Kaohsiung, Taiwan; ^14^Doctoral Degree Program, Department of Public Health, College of Health Sciences, Kaohsiung Medical University, Kaohsiung, Taiwan; ^15^Division of Pulmonary and Critical Care Medicine, Department of Internal Medicine, Kaohsiung Medical University Hospital, Kaohsiung Medical University, Kaohsiung, Taiwan; ^16^Department of Pediatrics, Kaohsiung Medical University Hospital, Kaohsiung Medical University, Kaohsiung, Taiwan; ^17^Division of Gastroenterology, Department of Internal Medicine, Kaohsiung Medical University Hospital, Kaohsiung Medical University, Kaohsiung, Taiwan

**Keywords:** synergistic effect, air pollutants, climate factors, obstructive lung disease, generalized additive model

## Abstract

**Background:**

Chronic Obstructive lung diseases (COPD) are complex conditions influenced by various environmental, lifestyle, and genetic factors. Ambient air pollution has been identified as a potential risk factor, causing 4.2 million deaths worldwide in 2016, accounting for 25% of all COPD-related deaths and 26% of all respiratory infection-related deaths. This study aims to evaluate the associations among chronic lung diseases, air pollution, and meteorological factors.

**Methods:**

This cross-sectional study obtained data from the Taiwan Biobank and Taiwan Air Quality Monitoring Database. We defined obstructive lung disease as patients with FEV1/FVC < 70%. Descriptive analysis between spirometry groups was performed using one-way ANOVA and the chi-square or Fisher’s exact test. A generalized additive model (GAM) was used to evaluate the relationship between SO_2_ and PM_2.5_/PM_10_ through equations and splines fitting.

**Results:**

A total of 2,635 participants were enrolled. Regarding environmental factors, higher temperature, higher relative humidity, and lower rainfall were risk factors for obstructive lung disease. SO_2_ was positively correlated with PM_10_ and PM_2.5_, with correlation coefficients of 0.53 (*p* < 0.0001) and 0.52 (p < 0.0001), respectively. Additionally, SO_2_ modified the relative risk of obstructive impairment for both PM_10_ [*β* coefficient (*β*) = 0.01, *p* = 0.0052] and PM_2.5_ (*β* = 0.01, *p* = 0.0155). Further analysis per standard deviation (per SD) increase revealed that SO_2_ also modified the relationship for both PM_10_ (*β* = 0.11, *p* = 0.0052) and PM_2.5_ (*β* = 0.09, *p* = 0.0155). Our GAM analysis showed a quadratic pattern for SO_2_ (per SD) and PM_10_ (per SD) in model 1, and a quadratic pattern for SO_2_ (per SD) in model 2. Moreover, our findings confirmed synergistic effects among temperature, SO_2_ and PM_2.5_/PM_10_, as demonstrated by the significant associations of bivariate (SO_2_ vs. PM_10_, SO_2_ vs. PM_2.5_) thin-plate smoothing splines in models 1 and 2 with obstructive impairment (*p* < 0.0001).

**Conclusion:**

Our study showed high temperature, humidity, and low rainfall increased the risk of obstructive lung disease. Synergistic effects were observed among temperature, SO_2_, and PM_2.5_/PM_10_. The impact of air pollutants on obstructive lung disease should consider these interactions.

## 1. Introduction

Obstructive lung diseases such as asthma, chronic obstructive pulmonary disease (COPD), and bronchiectasis are complex heterogeneous diseases resulting from interactions among environmental, lifestyle, and genotype factors. In 2015, around 358.2 and 174.5 million individuals worldwide had asthma and COPD, respectively, and 0.4 and 3.2 million people died from the diseases (1). The high prevalence and mortality associated with obstructive lung disease result in significant medical and social costs (2, 3) and therefore it is crucial to determine the risk factors and comorbidities that cause obstructive lung disease.

Ambient air pollution has been identified as a potential risk factor for obstructive lung disease. Air pollution is a mixture of hazardous substances, including particulate matter (PM_10_, PM_2.5_), sulfur dioxide (SO_2_), nitrogen monoxide (NO), nitrogen dioxide (NO_2_), nitrogen oxides (NO_x_), carbon monoxide (CO), and ozone (O_3_). Aerosol-like air pollutants are transported to the alveoli by inhalation, and PM is subsequently deposited in the respiratory tract. These air pollutants can induce the release of inflammatory mediators and lead to the development of obstructive lung disease. Previous studies have revealed associations between exposure to air pollutants and daily admissions for COPD (4) and increased mortality and morbidity (5, 6). In 2016, ambient air pollution was reported to cause 4.2 million deaths worldwide, including 25% of all COPD deaths and 26% of all respiratory infection-related deaths (7, 8). Ambient air pollution has also been associated with cardiovascular (9, 10) and central nervous system diseases (11). Furthermore, air pollution is correlated with meteorological factors (12). A previous study demonstrated an additive interaction between high temperature and air pollution (13), and another study found that a decrease in lung function was related to high temperature and humidity (14).

Air pollution usually contains many harmful components, and interactions between these components are possible. For example, Yun et al. found a synergistic effect between PM_10_ and SO_2_. In their study, cell damage and apoptosis occurred at low exposure to both PM_10_ and SO_2_, however these effects were not observed when exposed to either PM_10_ or SO_2_ alone at the same concentration (15). In addition, Ku et al. reported that low exposure to both PM_2.5_ and SO_2_ could lead to neurodegeneration (16). Moreover, interactions between fine particles with NO_2_ or O_3_ have also been associated with adverse effects such as cardiovascular diseases (17, 18) and respiratory diseases (19), as well as an increased risk of preterm birth (20). Taken together, interactions between air pollutants can affect health even at a low concentrations, and therefore it is important to understand the synergistic impact of air pollutants on health.

In this study, we aimed to evaluate the relationships among chronic lung diseases, air pollution, meteorological factors and anthropometric indices, and also the synergistic effect of SO_2_ and PM_2.5_/PM_10_. We hypothesized that exposure to SO_2_ and PM_2.5_/PM_10_ air pollution may be associated with lower lung function and higher prevalence of obstructive lung disease, even at relatively lower concentrations of PM_2.5_ and PM_10._

## 2. Materials and methods

### 2.1. Data source and study population

This cross-sectional study used data from two large databases: the Taiwan Biobank (TWB) and the Taiwan Air Quality Monitoring Database (TAQMD), both of which were obtained from the Taiwan Environmental Protection Administration (TEPA). The Taiwan Biobank (TWB) is the largest biobank in Taiwan, consisting of biological samples and associated data collected from volunteers aged between 30 and 70 years old who do not have a history of cancer. Prior to participation, every individual provided informed consent and underwent a face-to-face comprehensive interview, physical examination, blood sampling, and completed a questionnaire covering personal information and lifestyle factors. These procedures ensured that a detailed and comprehensive set of data could be collected for analysis, contributing to the understanding of health and disease in the Taiwanese population. We used data from 74 air quality monitoring stations located throughout Taiwan, as recorded by the TAQMD on a daily basis. The TAQMD was established by the Executive Yuan of the Taiwan Environmental Protection Administration, and is comprised of daily air pollutant concentration data at the study period of data collection. PM_2.5_ and PM_10_ were detected by β-ray attenuation method, SO_2_ was detected by ultraviolet fluorescence method, CO was determined by nondispersive infrared method, O_3_ was calculated by ultraviolet absorption method, NO_x_ was detected by chemiluminescence method. All air pollutant data is stored in the cloud every hour for free. The average concentrations of air pollutants in a selected year were obtained before analysis.

By utilizing both the TWB and TAQMD, we were able to determine the nearest air quality monitoring station to the residential addresses of the participants using a three-step procedure. First, we used Google geocoding to determine the exact geoposition of each residential address. Second, we determined the interpolation point between each residential address and the nearest air quality monitoring station. Lastly, we selected data from the air quality monitoring station recorded during the year leading up to the survey date and calculated the average values of air pollutants including PM_2.5_, PM_10_, CO, NO, NO_2_, NO_x_, SO_2_, and O_3_ for the chosen year (21).

### 2.2. Variables

The following variables were recorded: demographic characteristics including age, gender, smoking and alcohol consumption; anthropometric parameters including height, weight, body mass index (BMI), body adiposity index (BAI), and body roundness index (BRI); comorbidities including hypertension, type 2 diabetes, renal failure, metabolic syndrome, and coronary artery disease; region of Taiwan, including northern, central, and southern regions; and meteorological factors including temperature (in Celsius), relative humidity (in percentage), and rainfall (in millimeters).

### 2.3. Lung function status

Pulmonary function parameters including forced expiratory volume in one second (FEV1), forced vital capacity (FVC), FEV1/FVC% ratio, FVC-predicted value, and FEV1-predicted value, were recorded in the TWB. Technicians used MicroLab spirometers and Spida 5 software (Micro Medical Ltd., Rochester, Kent, UK) (22) to perform spirometry measurements. Obstructive lung diseases including asthma, COPD, and bronchiectasis were defined as patients with FEV1/FVC < 70%, according to the American Thoracic Society and European Respiratory Society guidelines.

### 2.4. Statistical analysis

We used one-way ANOVA and the chi-square or Fisher’s exact tests as appropriate. Multinomial logistic regression was used to estimate crude odds ratios (ORs) and 95% confidence intervals (CIs). Stepwise multinomial logistic regression was used to calculate adjusted ORs and 95% CIs. In addition, for the factors showing a significant association in the crude analysis, estimated adjusted ORs and 95% CIs were further used to evaluate associations between covariant factors and obstructive lung disease. Pearson’s correlation analysis was used to evaluate the relationships between variables (temperature, relative humidity, rainfall, PM_10_, PM_2.5_, and SO_2_). As correlations between SO_2_ and PM_2.5_ and SO_2_ and PM_10_ were found, a generalized additive model (GAM) was further used to evaluate the relationships between SO_2_ and PM_2.5_ and SO_2_ and PM_10_ to fit equations and splines, and to explore linear and nonlinear effects of SO_2_ and PM_2.5_ or PM_10_ on the outcomes of obstructive impairment. All data analyses were performed using SAS software version 9.4 (SAS Institute Inc., Cary, NC, USA).

## 3. Results

### 3.1. Profiles of the participants

The mean age of the 2,635 enrolled participants was 49.80 ± 10.53 years. Of these participants, 1,225 (46.5%) were men, and 1,410 (53.5%) were women. The participants were stratified into two groups according to lung function test results: the control group (normal spirometry group) and chronic lung disease group (obstructive impairment). Overall, 72.2% (1902/2635) of the participants were classified into the control group, and 27.8% (733/2635) were classified into the chronic lung disease group. Propensity score matching (1:2) was performed to balance the baseline characteristics between the two groups. Table 1 shows the results of baseline characteristics before and after propensity score matching.

**Table 1:** Descriptive statistics of the demographic, laboratory, meteorological factors, and air pollutants.

	Total	Obstructive impairment (2)	Normal spirometry (1)	*p*	Normal spirometry (1) (1:2 matching)*	*p*
*n*	2,635	733	1902		1,466	
FEV10_PRED, mean (SD)	84.89 (22.35)	58.42 (18.31)	95.09 (13.73)	<0.0001	95.13 (13.54)	<0.0001
≥80%	1747 (66.3)	80 (10.9)	1,667 (87.6)		1,291 (88.1)	
50–80%	639 (24.3)	408 (55.7)	231 (12.1)		171 (11.7)	
30–50%	204 (7.7)	200 (27.3)	4 (0.2)		4 (0.3)	
<30%	45 (1.7)	45 (6.1)	0 (0.0)	<0.0001	0 (0.0)	<0.0001
Age (years), mean (SD)	49.80 (10.53)	50.56(10.68)	49.51 (10.46)	0.0216	50.51 (10.64)	0.9220
30–39	587 (22.3)	149 (20.3)	438 (23.0)		306 (20.9)	
40–49	710 (26.9)	186 (25.4)	524 (27.5)		359 (24.5)	
40–59	816 (31.0)	231 (31.5)	585 (30.8)		466 (31.8)	
≥60	522 (19.8)	167 (22.8)	355 (18.7)	0.0632	335 (22.9)	0.9713
Sex, *n* (%)						
Male	1,225 (46.5)	322 (43.9)	903 (47.5)		654 (44.6)	
Female	1,410 (53.5)	411 (56.1)	999 (52.5)	0.1019	812 (55.4)	0.7615
Monitoring region, *n* (%)						
Northern region	494 (18.7)	182 (24.8)	312 (16.4)		312 (21.3)	
Central region	529 (20.1)	139 (19.0)	390 (20.5)		287 (19.6)	
Southern region	1,612 (61.2)	412 (56.2)	1,200 (63.1)	<0.0001	867 (59.1)	0.1691
Smoking, *n* (%)						
None	1917 (72.8)	535 (73.0)	1,382 (72.7)		1,086 (74.1)	
Current and former	718 (27.2)	198 (27.0)	520 (27.3)	0.8657	380 (25.9)	0.5836
Alcohol consumption, *n* (%)						
None and sometimes	2,371 (90.0)	660 (90.0)	1711 (90.0)		1,323 (90.2)	
Current and quit	264 (10.0)	73 (10.0)	191 (10.0)	0.9493	143 (9.8)	0.8792
Anthropometric parameter, mean (SD)						
Height (cm)	162.94 (8.25)	162.52 (8.05)	163.09 (8.32)	0.1114	162.32 (8.14)	0.5725
Weight (kg)	64.38 (12.05)	64.08 (11.6)	64.49 (12.22)	0.4324	63.91 (11.99)	0.7482
Body mass index mean (kg/m^2^)	24.14 (3.4)	24.16 (3.32)	24.13 (3.44)	0.8202	24.15 (3.45)	0.9286
Body adiposity index	28.5 (3.88)	28.75 (3.79)	28.40 (3.92)	0.0369	28.75 (3.92)	0.9799
Body roundness index	3.71 (1.11)	3.74 (1.08)	3.70 (1.12)	0.4180	3.73 (1.13)	0.8682
Comorbidities, *n* (%)						
Hypertension	275 (10.4)	78 (10.6)	197 (10.4)	0.8310	161 (11.0)	0.8086
Diabetes mellitus type 2	120 (4.6)	43 (5.9)	77 (4.0)	0.0449	60 (4.1)	0.0635
Renal failure	4 (0.2)	1 (0.1)	3 (0.2)	0.8998	3 (0.2)	0.7234
Metabolic syndrome	475 (18.0)	136 (18.6)	339 (17.8)	0.6620	277 (18.9)	0.8469
Coronary artery disease	27 (1.0)	6 (0.8)	21 (1.1)	0.5143	19 (1.3)	0.3194
Meteorological factors, mean (SD)						
Temperature (°C)	24.33 (0.75)	24.41 (0.84)	24.31 (0.72)	0.0016	24.27 (0.75)	0.0001
Relative humidity (%)	74.28 (2.45)	74.51 (2.37)	74.20 (2.47)	0.0028	74.25 (2.49)	0.0158
Rainfall (mm/day)	0.22 (0.05)	0.21 (0.05)	0.22 (0.05)	0.0039	0.22 (0.05)	0.0001
Air pollution factors, median (IQR)						
PM_10_ (μg/m^3^)	68.12 (17.2)	65.72 (17.51)	69.05 (16.99)	<0.0001	67.74 (17.69)	0.0113
PM_2.5_ (μg/m^3^)	37.72 (10.8)	35.88 (10.74)	38.44 (10.74)	<0.0001	37.47 (11.15)	0.0014
CO (ppm)	0.44 (0.18)	0.45 (0.20)	0.44 (0.17)	0.3033	0.45 (0.18)	0.7156
NO (ppb)	4.09 (3.83)	4.31 (4.29)	4.00 (3.64)	0.0666	4.19 (4.08)	0.5400
NO_2_ (ppb)	14.86 (5.6)	14.76 (6.45)	14.9 (5.23)	0.5875	14.83 (5.72)	0.8188
NO_X_ (ppb)	18.93 (8.71)	19.06 (9.94)	18.88 (8.19)	0.6431	19.0 (9.08)	0.8936
O_3_ (ppb)	30.97 (3.85)	31.04 (4.04)	30.94 (3.78)	0.5466	30.89 (3.88)	0.3957
SO_2_ (ppb)	3.63 (1.19)	3.70 (1.39)	3.61 (1.09)	0.0809	3.57 (1.15)	0.0265

The two groups were propensity-score matched (1:2) for baseline characteristics of age categories, sex, live region, Smoke, Drink, BMI, BAI and BRI. Air pollution factors were analyzed using independent *t-*test to compare the obstructive impairment group with the comparison group of normal spirometry.

There were no significant differences in age, gender, smoking, alcohol consumption, anthropometric factors and comorbidities, including hypertension, type 2 diabetes mellitus, renal failure, metabolic syndrome, and coronary artery disease between the two groups. Regarding meteorological factors, higher temperature, higher relative humidity, and lower rainfall were risk factors for obstructive lung disease. In addition, we found that exposure to SO_2_ in the environment increased the impact on patients with obstructive lung disease, whereas PM_2.5_ and PM_10_ decreased the impact (Table 1).

### 3.2. Correlations among meteorological factors and SO_2_, PM_2.5_/PM_10_

We found that SO_2_ was positively correlated with PM_10_ and PM_2.5_, with correlation coefficients of 0.53 (*p* < 0.0001) and 0.52 (*p* < 0.0001), respectively (Table 2). In addition, PM_10_ and PM_2.5_ were also positively correlated (correlation coefficient = 0.69, *p* < 0.0001).

**Table 2:** Pearson correlation coefficients and *p*-values.

	Temperature (°C)	*P-* value	Relative humidity (%)	*P-* value	Rainfall (mm/day)	*P-* value	PM_10_ (μg/m^3^)	*P-* value	PM_2.5_ (μg/m^3^)	*P-* value	SO_2_ (ppb)
Temperature (°C)	1.00										
Relative humidity (%)	−0.15	<0.0001	1.00								
Rainfall (mm/day)	−0.37	<0.0001	−0.18	<0.0001	1.00						
PM_10_ (μg/m^3^)	0.19	<0.0001	−0.37	<0.0001	0.14	<0.0001	1.00				
PM_2.5_ (μg/m^3^)	0.28	<0.0001	−0.37	<0.0001	0.08	0.0003	0.69	<0.0001	1.00		
SO_2_ (ppb)	0.08	<0.0001	−0.33	<0.0001	0.21	<0.0001	0.53	<0.0001	0.52	<0.0001	1.00

### 3.3. Associations among obstructive lung disease, meteorological factors and SO_2_, PM_2.5_/PM_10_

To further determine whether SO_2_ modified the relationship of PM_10_ or PM_2.5_ with the relative risk of obstructive impairment, beta coefficients with standard error [β (SE)] and *p-*values for interaction were calculated. The results showed that SO_2_ modified the relationship of both PM_10_ (*β* = 0.01, *p* = 0.0052) and PM_2.5_ (*β* = 0.01, *p* = 0.0155) with the relative risk of obstructive impairment (Table 3). Analysis of per standard deviation (per SD) increase also showed that SO_2_ modified the relationship of both PM_10_ (*β* = 0.11, *p* = 0.0052) and PM_2.5_ (*β* = 0.09, *p* = 0.0155). Table 3 shows the crude ORs of meteorological factors and SO_2_, PM_2.5_/PM_10_. Compared with the control group, the obstructive impairment group was associated with higher temperature, higher relative humidity, and lower rainfall, and also exposure to a higher level of SO_2_ and lower levels of PM_2.5_ and PM_10_. Interactions were also identified between SO_2_ and PM_2.5_/PM_10_ (Table 3). Model 1 showed that the independent predictive factors were temperature (OR = 1.24; 95% CI = 1.09–1.41; *p* = 0.0009), relative humidity (OR = 1.05; 95% CI = 1.01–1.10; *p* = 0.0160), rainfall (OR = 0.08; 95% CI = 0.01–0.68; *p* = 0.0202), PM_10_ (OR = 0.99; 95% CI = 0.98–0.99; *p* < 0.001), and SO_2_ (OR = 1.25; 95% CI = 1.14–1.36; *p* < 0.001). Model 2 showed that the independent predictive factors were temperature (OR = 1.31; 95% CI = 1.15–1.49; *p* < 0.001), relative humidity (OR = 1.04; 95% CI = 1.00–1.09; *p* = 0.0372), rainfall (OR = 0.08; 95% CI = 0.01–0.67; *p* = 0.0197), PM_2.5_ (OR = 0.97; 95% CI = 0.96–0.98; *p* < 0.001), and SO_2_ (OR = 1.28; 95% CI = 1.17–1.39; *p* < 0.001).

**Table 3:** Predicted obstructive impairment by crude and multiple logistic regression model.

			PM_10_ or PM_2.5_ by SO2	Model 1		Model 2		PM_10_ or PM_2.5_ by SO2
	Crude OR (95%CI)	*P-*value	β (SE), P for interaction	Adjusted OR (95%CI)	*P-*value	Adjusted OR (95%CI)	*P-*value	Adjusted β (SE), *P* for interaction
Temperature (°C)	1.26 (1.12–1.41)	0.0001		1.24 (1.09–1.41)	0.0009	1.31 (1.15–1.49)	<0.0001	
Relative humidity (%)	1.05 (1.01–1.08)	0.0160		1.05 (1.01–1.10)	0.0160	1.04 (1.00–1.09)	0.0372	
Rainfall (mm/day)	0.03 (0.00–0.18)	0.0001		0.08 (0.01–0.68)	0.0202	0.08 (0.01–0.67)	0.0197	
PM_10_ (μg/m^3^)	0.99 (0.99–0.999)	0.0115	0.01 (0.00), 0.0052	0.99 (0.98–0.99)	<0.0001			0.00 (0.00), 0.3423
PM_2.5_ (μg/m^3^)	0.99 (0.98–0.99)	0.0015	0.01 (0.00), 0.0155			0.97 (0.96–0.98)	<0.0001	0.05 (0.05), 0.3423
SO_2_ (ppb)	1.08 (1.01–1.16)	0.0268		1.25 (1.14–1.36)	<0.0001	1.28 (1.17–1.39)	<0.0001	
Per SD increasing								
Temperature (°C)	1.19 (1.09–1.30)	0.0001		1.18 (1.07–1.3)	0.0009	1.22 (1.11–1.35)	<0.0001	
Relative humidity (%)	1.12 (1.02–1.22)	0.0160		1.13 (1.02–1.25)	0.0160	1.11 (1.01–1.23)	0.0372	
Rainfall (mm/day)	0.84 (0.76–0.92)	0.0001		0.88 (0.80–0.98)	0.0202	0.88 (0.80–0.98)	0.0197	
PM_10_ (μg/m^3^)	0.90 (0.82–0.98)	0.0115	0.11 (0.04), 0.0052	0.79 (0.71–0.88)	<0.0001			−0.00 (0.00), 0.5086
PM_2.5_ (μg/m^3^)	0.87 (0.80–0.95)	0.0015	0.09 (0.04), 0.0155			0.73 (0.65–0.81)	<0.0001	−0.03 (0.04), 0.5086
NO_2_ (ppb)	0.99 (0.91–1.08)	0.8187		1.09 (0.86–1.37)	0.4895	1.06 (0.83–1.34)	0.6488	
O_3_ (ppb)	1.04 (0.95–1.13)	0.3958		1.03 (0.86–1.23)	0.7584	1.00 (0.83–1.20)	0.9979	
SO_2_ (ppb)	1.10 (1.01–1.20)	0.0268		1.30 (1.17–1.44)	<0.0001	1.34 (1.21–1.48)	<0.0001	

To determine whether SO_2_ modified the relationship of PM_10_ or PM_2.5_ with the relative risk of obstructive impairment, β (standard error, SE) and *P-*value for interaction were calculated.

### 3.4. Interactions among obstructive lung disease with SO_2_ and PM_2.5_ or PM_10_

**Figure 1:**
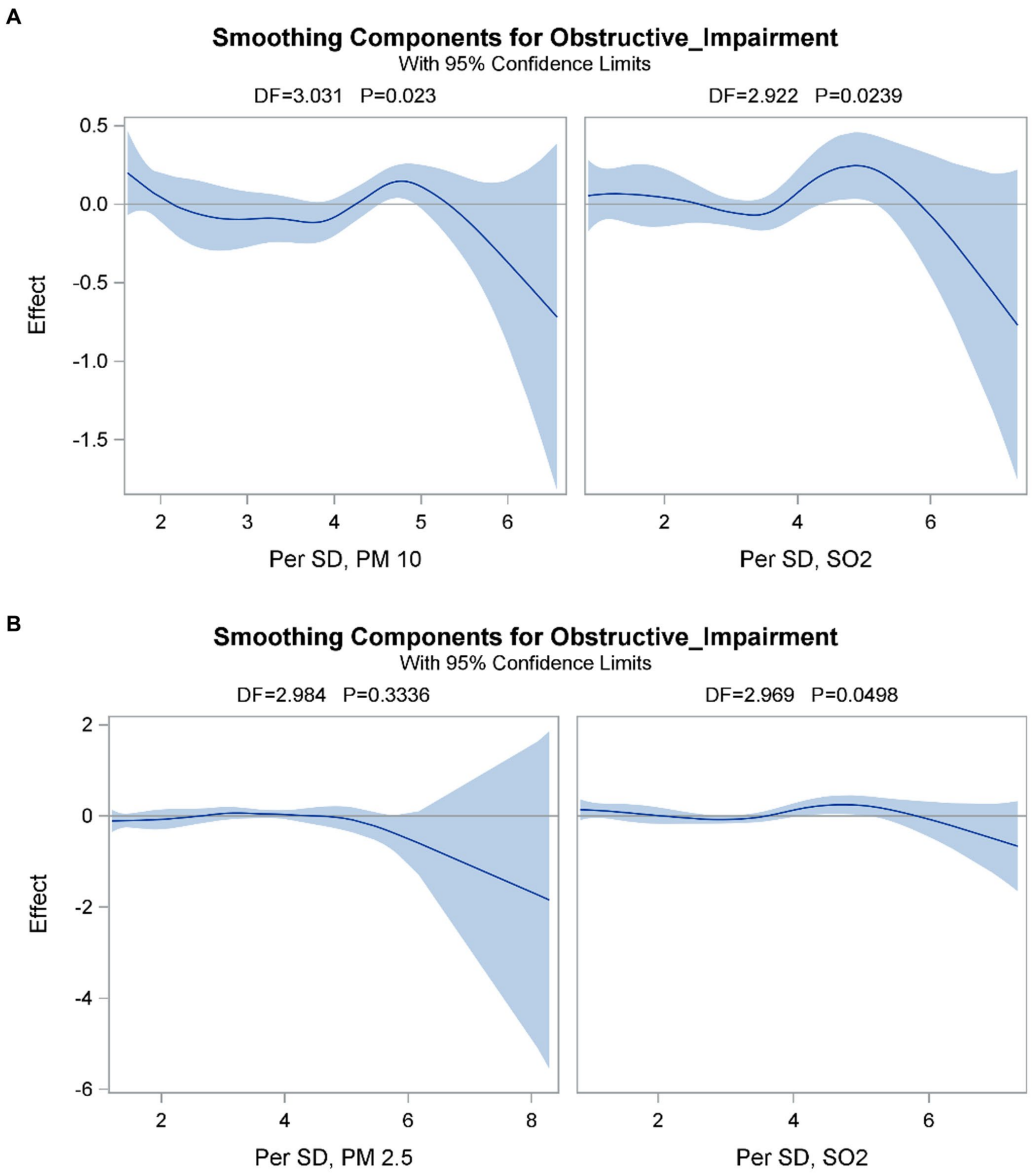
Partial prediction of A) SO_2_ (Per SD) and PM10 (Per SD) in model 1 and B) SO_2_ (Per SD) and PM2.5 (Per SD) on the risk of obstructive impairment. A semiparametric model was performed by using the parametric effects of temperature (°C), relative humidity (%) and Rainfall (mm/day) as the linear part of the model. Obstructive impairment was associated with a quadratic pattern for the SO_2_ (Per SD) and PM10 (Per SD) in model 1 and a quadratic pattern for the SO_2_ (Per SD) but not PM2.5 (Per SD) in model 2.

**Figure 2:**
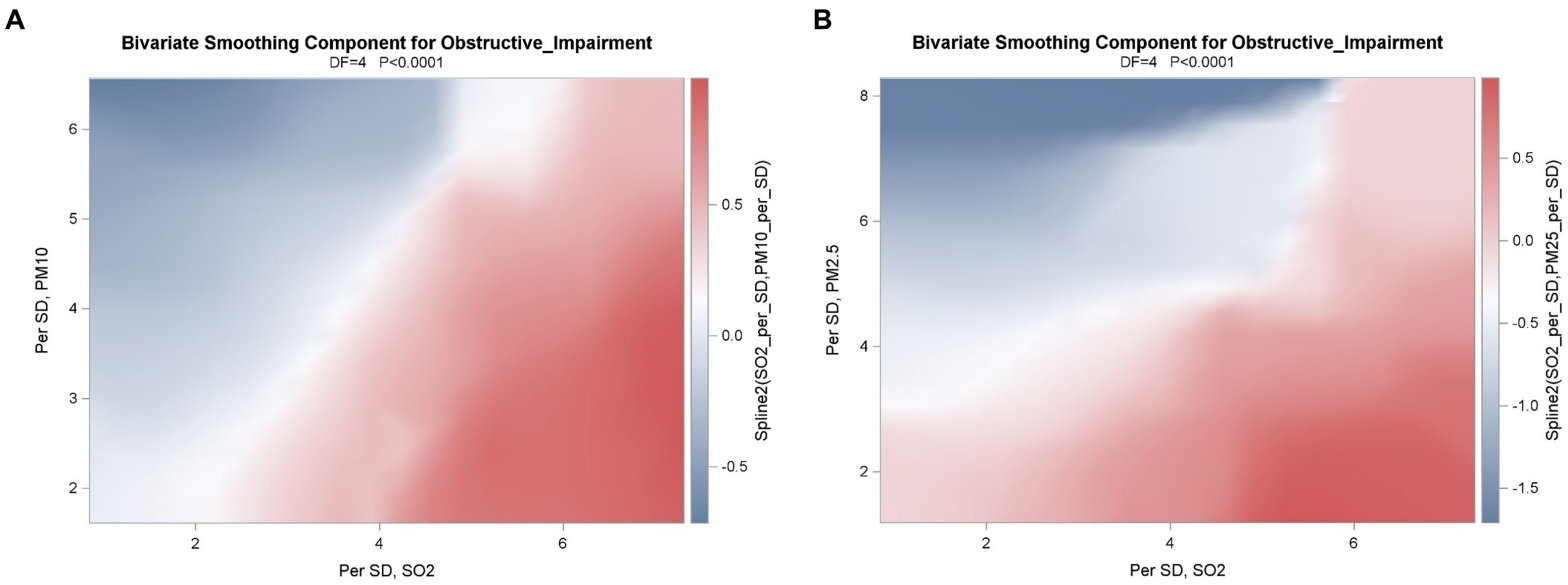
Correlations between A) SO_2_ (Per SD) and PM10 (Per SD) in model 1 and B) SO_2_ (Per SD) and PM2.5 (Per SD) in model 2 of obstructive impairment were applied by the use of a generalized additive model (GAM), a smoothing spline nonparametric model. A semiparametric model was performed by using the parametric effects of temperature (°C), relative humidity (%) and Rainfall (mm/day) as the linear part of the model. The graphic suggests that there was an interaction, a diagonal pattern in model 1 and model 2, on the risk of obstructive impairment.

The GAM (Figure 1) showed that obstructive impairment was associated with a quadratic pattern for SO_2_ (per SD) and PM_10_ (per SD) in model 1, and a quadratic pattern for SO_2_ (per SD) but not PM_2.5_ (per SD) in model 2. We also found that the bivariate thin-plate smoothing spline in models 1 and 2 were significantly associated with obstructive impairment (*p* < 0.0001) (Table 4). In addition, bivariate smoothing of SO_2_, PM_10_ and PM_2.5_ showed evidence of the risk of obstructive impairment (Figures 2A,B). A semiparametric model was generated using the parametric effects of temperature (°C), relative humidity (%) and rainfall (mm/day) as the linear part of the model.

**Table 4:** Predicted obstructive impairment by generalized additive model, a smoothing spline nonparametric model.

	DF	Sum of squares	Chi-square	*P-*value
Model 1				
Spline (Per SD, PM_10_)	3.03	9.59	9.59	0.0230
Spline (Per SD, SO_2_)	2.92	9.30	9.30	0.0239
Bivariate thin-plate smoothing spline*				
Spline2(SO_2_ per SD, PM_10_ per SD)	4.00	37.19	37.19	<0.0001
Model 2				
Spline (Per SD, PM_2.5_)	2.98	3.38	3.38	0.3336
Spline (Per SD, SO_2_)	2.97	7.77	7.77	0.0498
Bivariate thin-plate smoothing spline*				
Spline2 (SO_2_ per SD, PM_2.5_ per SD)	4.00	48.04	48.04	<0.0001

A semiparametric model was performed by using the parametric effects of temperature (°C), relative humidity (%) and Rainfall (mm/day) as the linear part of the model. *Fits a bivariate thin-plate smoothing spline with SO_2_ per SD and PM_10_ per SD or SO_2_ per SD and PM_2.5_ per SD and with DF = 4.

## 4. Discussion

In this study, we analyzed 2,635 participants in the TWB and found that factors associated with a higher risk of obstructive lung disease included higher temperature, higher relative humidity, and lower rainfall. We also found that SO_2_ was strongly associated with obstructive lung disease, while PM_2.5_ and PM_10_ were not. Further analysis revealed that SO_2_ synergistically interacted with PM_2.5_ and PM_10_ to increase the risk of obstructive lung disease.

Overall, 27.8% of our study population had obstructive impairment. However, a previous study estimated that the prevalence of COPD in Taiwan was around 6.1% (23), with a prevalence of asthma of around 5.1% (24). The higher percentage of obstructive impairment in our study may be due to the presence of higher annual mean concentrations of air pollutants in southern Taiwan than in other areas (25, 26). In Table 1, we present the average air pollution levels based on a total of 2,635 observations, indicating the following values: PM_10_: 68.12 μg/m^3^, PM_2.5_: 37.72 μg/m^3^, SO_2_: 3.63 ppb, CO: 0.44 ppm, NO: 4.09 ppb, NO_2_: 14.86 ppb, NO_x_: 18.93 ppb. Furthermore, around 1,612 individuals, which accounts for 61.2% of the total, were from southern Taiwan. The findings align with those of our prior study (21). Fine particles play an essential role in the development of obstructive lung disease (25), and thus people exposed to higher concentrations of air pollution may have a higher prevalence of lung impairment.

We also found that people living in areas with a higher temperature, higher relative humidity In a previous study in Taiwan, Wu et al. reported a V/U shaped relationship between temperature and air pollutants (12), and a temperature between 24.3–24.9°C was associated with exposure to the lowest concentration of air pollutants. Thus, a higher or lower temperature may result in higher exposure to air pollution, which may then affect the development of obstructive lung disease. A study in New York City found that the risk of hospitalization due to respiratory diseases increased by 2.7% per °C above the threshold of 28.9°C on the same day (27). Another study in London revealed that the risk of respiratory diseases was related to admission when the temperature increased by 5.44% per °C above a threshold (23°C) with a lag of 0–2 days (28). Thus, a higher temperature appears to increase the risk of developing obstructive lung disease. When considering temperature and relative humidity, previous research has revealed a 0.7% decrease in FVC when there is a 5°C increase in the 3-day moving average temperature, and a 0.2% decrease in FVC when there is a 5% increase in the 7-day moving average relative humidity (14). Thermoregulation involves increasing cardiac output, cutaneous blood flow, and breathing rate. However, in conditions of high relative humidity evaporation by perspiration is limited, which creates physiological stress leading to dysfunction in respiratory function (29), especially in older people (30, 31). High temperature with high humidity has also been shown to affect thermoregulation and trigger bronchoconstriction (32). Thus, the risk of developing obstructive lung disease would increase under these conditions.

Our study also found that lower rainfall increased the risk of obstructive lung disease. A study conducted in Korea reported that the concentrations of air pollutants, including PM_10_ and NO_2_ were lower during rainfall compared to dry conditions (33). Another study in Korea revealed that pollutant (PM_10_, SO_2_, NO_2_, and CO) concentrations and rainfall intensity were significantly negatively correlated due to precipitation scavenging. Among those pollutants, PM_10_ was the most effectively scavenged by rain (34). In addition, a study in Spain reported a washout effect, with a 20% reduction in the number of particles during rainfall with an intensity of over 3.2 ± 1.5 mm/h (35). Thus, concentrations of air pollutants decrease due to a washout effect during rainfall, and consequently lower rainfall may be associated with a higher risk of obstructive lung disease.

Another finding of this study is that exposure to a higher level of SO_2_ and lower levels of PM_2.5_ and PM_10_ increased the risk of obstructive lung disease. SO_2_ is produced from volcanoes gas, burning fuel and industrial production processes (36–38). Exposure to SO_2_ has been shown to affect the respiratory tract and cause oxidative stress and DNA damage, which would further damage the lungs (39). Several studies have revealed a relationship between SO_2_ exposure and respiratory diseases (40–42). Goudarzi et al. concluded that a higher SO_2_ concentration was associated with an increased relative risk of hospital admission for respiratory diseases (43).

Particulate matter can be generated from soil dust, road traffic, industry, and fuel combustion, and it is a crucial indicator of the health effects of air pollution (44, 45). Several studies have discussed the relationship between PM and lung function change and respiratory diseases (12, 46, 47). Penttinen et al. reported a decrease in average evening peak expiratory flow by 1.14 L/min when the average concentration of PM_2.5_ increased by one interquartile (1.3 μg/m^3^) in a 5-day average (48). In addition, Downs et al. found significant negative associations between a lower concentration of PM_10_ and worsening lung function. They found that the annual decline in lung function with regards to FEV1 and FEF25–75 decreased by 9 and 16%, respectively, with a 10 μg/m^3^ reduction in PM_10_ over an 11-year period (49). Thus, higher concentrations of SO_2_ and PM appear to increase the risk of worsening lung function and developing obstructive lung disease. In our study, lower levels of PM_2.5_ and PM_10_ increased the risk of developing obstructive lung disease, which is contrast to most of previous studies. That is because, we found that there was a synergistic effect between SO_2_ and PM_2.5_/PM_10_. Yun et al. found that synergistic injury in terms of cell survival and apoptosis occurred under low concentrations of PM_10_ and SO_2_ (15). The proposed mechanism was that PM_10_ and SO_2_ synergistic induced cytotoxicity of radical oxygen species production and nuclear factor kappa B (NF-κB) activation (15, 50). Thus, the synergistic effect could increase the risk of respiratory diseases, even with low concentrations of the air pollutants. The synergistic effect could also explain our finding that a higher level of SO_2_ and lower levels of PM_2.5_ and PM_10_ increased the risk of obstructive lung disease. Furthermore, our results also showed that high SO_2_ exposure could affect lower concentrations of PM_2.5_ and PM_10_ with similar patterns (Figures 1, 2). These interesting findings indicate that SO_2_ could trigger PM_2.5_ and PM_10_, and that the interaction between SO_2_ and PM_2.5_/PM_10_ may play a vital role in developing obstructive lung disease.

Although our study is the first to comprehensively investigate the associations among obstructive lung disease (classified by lung function), air pollution, and meteorological factors, several limitations should be acknowledged. First, the design of this study was cross-sectional. Determining the progression of lung function and obstructive lung disease over time is complex, and further prospective studies are needed to elucidate the causal effects. Second, lung function assessments were used to identify chronic lung disease, and follow-up checkups are required to further evaluate the progression of the disease. Third, the TWB does not contain information regarding occupational exposure to toxic substances. Some poisonous substances may influence lung function, however we could not analyze this. Finally, because the participant’s residential address was used as the air pollutant exposure point, we did not include all factors affecting lung function, such as personal exposure, travel exposure, and indoor air quality. This may have led to underestimation of the risk of lung function impairment and the association with obstructive lung disease.

## 5. Conclusion

Compared with the normal spirometry group, we found that factors associated with a higher risk of obstructive lung disease included a higher temperature, higher relative humidity, and lower rainfall. Furthermore, we identified interactions and synergistic effects among SO_2_ and PM_2.5_/PM_10_. These findings could explain why a higher level of SO_2_ and lower levels of PM_2.5_/PM_10_ were associated with a higher risk of obstructive lung disease. Our findings also highlight the importance of interactions between air pollutants. We suggest that the synergistic effects of air pollutants should be considered when investigating the actual impact on developing obstructive lung disease.

## Data availability statement

The original contributions presented in the study are included in the article/supplementary material, further inquiries can be directed to the corresponding author.

## Ethics statement

The studies involving humans were approved by Institutional Review Board-1, Kaohsiung Medical University Chung-Ho-Memorial Hospital [KMUHIRB-E(I)-20180242]. The studies were conducted in accordance with the local legislation and institutional requirements. Written informed consent for participation was not required from the participants or the participants’ legal guardians/next of kin because this cross-sectional study obtained data from the Taiwan Biobank and Taiwan Air Quality Monitoring Database.

## Author contributions

P-SC and S-CC: conceptualization and supervision. T-YC and D-WW: writing original draft and formal analysis. H-PT: methodology and supervision. C-WW: investigation and formal analysis. C-HH and C-HL: investigation and supervision. C-SH: writing review and editing. C-HK: supervision. All authors have read and agreed to the published version of the manuscript.

## Funding

This work was supported partially by the Research Center for Precision Environmental Medicine, Kaohsiung Medical University, Kaohsiung, Taiwan from The Featured Areas Research Center Program within the framework of the Higher Education Sprout Project by the Ministry of Education (MOE) in Taiwan and by Kaohsiung Medical University Research Center Grants (KMU-TC112A01) and Kaohsiung Municipal Siaogang Hospital (S-110-05).

## Conflict of interest

The authors declare that the research was conducted in the absence of any commercial or financial relationships that could be construed as a potential conflict of interest.

## Publisher’s note

All claims expressed in this article are solely those of the authors and do not necessarily represent those of their affiliated organizations, or those of the publisher, the editors and the reviewers. Any product that may be evaluated in this article, or claim that may be made by its manufacturer, is not guaranteed or endorsed by the publisher.
